# The Role of Instructional Constraints Performed by Coaches on Tactical Behavior of Soccer Players: A Systematic Review

**DOI:** 10.5114/jhk/202057

**Published:** 2025-11-20

**Authors:** Cristiano Zarbato Morais, Júlio César da Silva Bispo, Lucas Shoiti Carvalho Ueda, Michel Milistetd, Juliano Fernandes da Silva, Paulo Henrique Borges

**Affiliations:** 1 Department of Physical Education, Federal University of Santa Catarina, Florianópolis, Brazil.

**Keywords:** feedback, ecological dynamics, coaching

## Abstract

Among strategies used by coaches in the training environment, feedback appears as a common coach practice. Regarding the collective characteristics of soccer, it is important to observe the game in the same scale. In the coaching process, players’ behavior can be constrained by coaches using feedback. This study aimed to investigate the role of feedback, performed by coaches, on the tactical behavior of soccer players. We searched in five databases: Scopus, Pubmed, Scielo, SportDiscus and Web of Science, following PRISMA-P guidelines. The PICOS strategy was used to establish eligibility criteria. Only quantitative studies written in English and published in peer reviewed journals were included. From the 1,149 articles found in the initial search, six were included in the review, and then were evaluated considering their methodological quality and risk of bias, through the quality index and the Joanna Briggs Institute (JBI) critical appraisal tools, respectively. Papers presented high heterogeneity regarding methods to apply feedback interventions and to assess the players’ tactical behavior. Differences were observed in the task proposed to players, varying from small-sided games to official size games (3 vs. 3 to 11 vs. 11). Despite these differences, similarities could be found regarding the use of notational analysis in half of the papers, and analysis through positional data in the other half. The use of instructional constraints before the game seems to bring more clarity on how to better coordinate collective actions, causing a positive effect on players’ tactical behavior.

## Introduction

During their intervention, coaches must contend with numerous elements in the training environment, including their relationships with managers, peers, and players, as well as the design of tasks to achieve their goals (Cushion et al., 2012; Petiot et al., 2021). Coaches can guide players' learning by manipulating task constraints, emphasizing certain aspects over others through operational and functional modifications to the task using verbal instruction, typically defined as feedback (Orth et al., 2019; Wood et al., 2023). The use of verbal instructions has significant effects on learning and performance of players (Davids et al., 2008; Klatt and Noël, 2020; Otte et al., 2020), in addition to being the coaches’ most practiced activity during training sessions (Hodges and Franks, 2002; Otte et al., 2020; Potrac et al., 2000).

From a classical perspective, rooted in behaviorism, feedback is defined as a way of conditioning behavior by providing information in a stimulus-response-reinforcement perspective and, within this logic, only interventions performed after the execution of an action would fit this concept (Panadero and Lipnevich, 2022). Consequently, feedback can be characterized by its shape and content and is commonly subdivided into intrinsic (players obtain information from the environment or their performance through their sensory system) and extrinsic (through the action of an external agent). Feedback is often divided into knowledge of results and knowledge of performance (Newell, 1991; Otte et al., 2020). At this point, there is a predominance of investigations into technical and physical dimensions, verifying the effect of feedback on the execution of a specific technique, performed out of the game context, with a focus on action efficiency (Bergmann et al., 2021; Pacheco et al., 2023; Silva and Drews, 2023).

However, when considering a different paradigm such as ecological dynamics which proposes that learning occurs through constant interaction among the individual, the environment, and the task they perform, feedback and/or instructions from the coach are defined as an instructional constraint related to the task (Davids et al., 2003; Otte et al., 2020). Those constraints, regardless of criteria used to observe the phenomena, can be classified by their type, content and moment in which they were carried out. As for the type, Otte et al. (2020) present seven categories in which instructional constraints can be classified, which are instructive (direct), task-oriented, question and answer, trial and error, video feedback, learning by model and learning by analogy. Regarding the content of the instructional constraint, it is necessary to be clear about the objective of this intervention, because even if directed at just one individual, that information can constrain the behavior of different elements involved in the system. Finally, regarding the moment, usually given before the game or training, it is named instruction, while if given after, it is named feedback.

In this view, feedback would not be used as a means of “feeding the chain”, but rather as a way of directing the players' attention to certain elements considered more appropriate to the coach's proposals. This perspective allows considering the nature of the game, observing it from the tactical standpoint, where the complexity and dynamics of multiple actions resulting from the game regulate the participants' behaviors within the system (Davids et al., 2013; Newell, 1986). Understanding that these actions are permeated by moments of unpredictability, the information arising from multiple interactions, together with existing constraints, influences and modifies players’ behaviors (Torrents et al., 2016). In this regard, the processes of exploration and discovery of how to solve problems presented in the training and/or in the game can be facilitated by the coach, using instructional constraints to better guide participants to the emergence of actions that meet the demands of the imposed tactical scenario (Davids et al., 2003).

The perception of soccer from the tactical standpoint meets the assumption that players share an environment permeated by cooperative-oppositive relationships (Gonzaga et al., 2014). In this context, players start from smaller-scale tactical actions, such as the individual micro-scale, moving on to coordinated group actions (meso-scales comprising two, three or four players), reaching the collective dynamics that integrate behaviors on a macro scale (Garganta and Gréhaigne, 2007; Passos et al., 2011). In this scenario, the coach intervention, through the information provided to players, plays an important role in their behavior, offering information from an external source to regulate interactions and actions performed within the playing environment (Sigrist et al., 2013).

The literature regarding feedback in soccer has shown strong focus on the technical dimension, based on the quality of motor responses (Chiviacowsky and Drews, 2014; Hicheur et al., 2020; Pacheco et al., 2023) and, thus, the instructive or direct type of feedback is the most common and usually applied in experimental protocols. However, the impact of these instructional constraints on tactical performance still needs further research.

Considering that soccer is a complex sport characterized by its interactions among different elements (Gonzaga et al., 2014), and that the use of structural and functional constraints affects players’ tactical behaviors (Los Arcos et al., 2025; Ueda et at., 2025), it is important to understand how instructional constraints in the training context modify these behaviors. Therefore, this systematic review aimed to investigate the role of instructional constraints, performed by coaches, on the tactical behavior of soccer players. It was hypothesized that the coaches’ interventions could help players improve their collective performance, once they could bring more information regarding the task, environment, and/or players’ performance, to support their coordination in training sessions and/or games.

## Methods

To begin the production of the review, on the 19^th^ of April, 2024, the protocol was registered on the Open Science Framework (OSF) platform (DOI: 10.17605/OSF.IO/GRBKT), establishing the procedures regarding the systematic search strategy, eligibility criteria, and the methods used to assess the quality of included studies. Following the recommendations of PRISMA-P (Moher et al., 2015), the protocol and writing of this systematic review were conducted.

### Information Sources and the Study Selection Process

The search in databases (Pubmed, Scielo, Scopus, SportDiscus and Web of Science) was performed on April 20, 2024. We conducted an extra search in the reference lists of the included articles, and also contacted via e-mail researchers considered experts in the field. To perform the search, the following strategy was used: (soccer OR football) AND (feedback OR augmented OR “instruction*” OR “attentional focus”) AND (performance OR learning OR knowledge OR tactical OR technical OR skill OR “motor control”). From the search, results were obtained as files under three different formats, “RIS”, “TXT” and “BIB”, which are commonly used to report their references. These files were inserted into the Rayyan web application for managing and simplifying the systematic review process.

To carry out the study selection, the PRISMA 2020 Flow Diagram for New Systematic Reviews (Page et al., 2021) was used as a guide. After the search was performed, and reports were uploaded to the Rayyan web application to exclude duplicates, two reviewers (reviewer 1 and 2) carried out the study selection, first considering the title and the abstract, and then the full text; in the event of disagreement a third reviewer was consulted. To complement the search, to find articles not covered by the strategy used, two additional strategies were applied: email contact with experts, and the manual search in the reference lists of included studies.

### Eligibility Criteria

The PICOS strategy (Methley et al., 2014) was used to establish the eligibility criteria. As previously established in the protocol, only full articles written in the English language and published in peer-reviewed journals were considered to be analyzed in this systematic review. No restrictions regarding time intervals were established. The PICOS strategy was also used to define the exclusion criteria.

Eligibility criteria:

- Population: soccer players, without any restrictions.
- Intervention: coaches’ feedback, without restrictions on the type and content, in the soccer context.
- Comparison/control: not applicable.
- Outcome measures: tactical behavior, considering every measure that could be related to the tactical domain, only in the deliberate practice context.
- Type of the study: articles published in peer-reviewed journals written in the English language, with a quantitative approach.

### Data Synthesis and Qualitative Analysis of the Studies

To characterize the included studies, the search used information regarding the authors, the year of publication, the country, the sample, the context (performance level), the feedback type (type of feedback used in the intervention), feedback moment (based on the intervention, in which moment feedback was used), the game format, measure of tactical behavior, and main outcomes (related to tactical elements).

To assess the risk of bias across studies, the PRISMA recommendations were followed by two reviewers using the Joanna Briggs Institute (JBI) critical appraisal tools for systematic reviews of cross-sectional studies (Moola et al., 2020). The methodological quality of included studies was assessed using an adapted version of the quality index (Downs and Black, 1998), which has been adopted in recent systematic reviews with correlated subjects (Praça et al., 2022; Ueda et al., 2023).

## Results

### Study Selection

**Figure 1:**
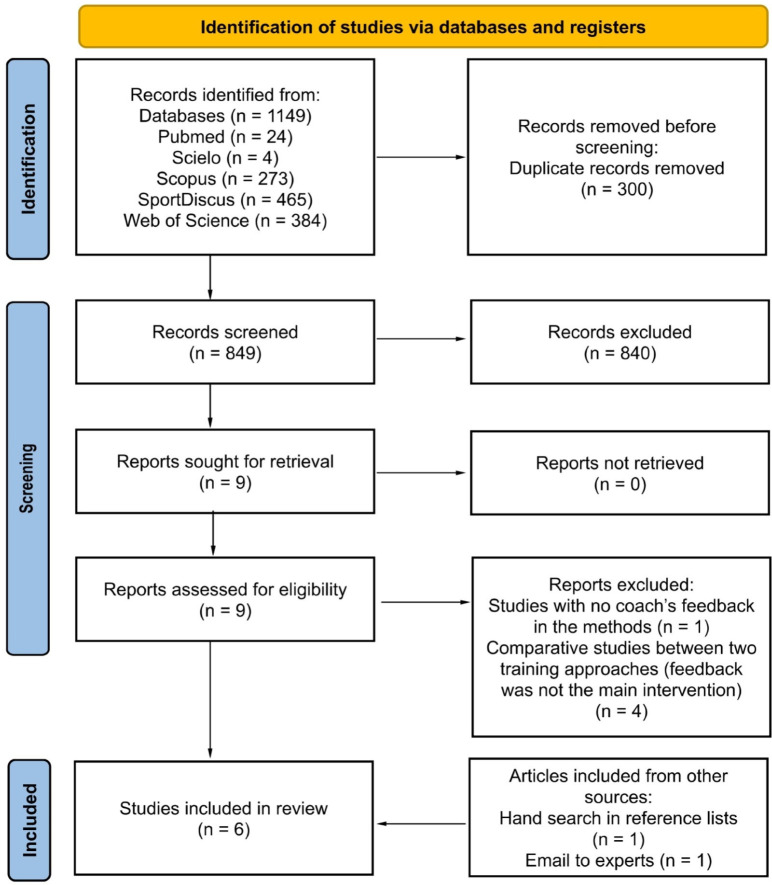
PRISMA flow diagram highlighting the selection process of studies (adapted from Page et al., 2021).

Figure 1 shows a summary of the process of studies’ selection and identification. The search in the databases resulted in a total of 1,149 references, and after the exclusion of duplicates, 849 studies were considered for further analysis. In the first stage of screening, 849 studies were assessed, and nine were found to be eligible to the next stage, where reviewers 1 and 2 read the full articles. At this stage, five studies were excluded, four for being comparative studies of two different training approaches, and one for not including coach feedback in the methods. Thus, at this stage, four studies satisfied the inclusion criteria and were considered to the qualitative analysis. As mentioned above, an additional search was conducted, where two methods were used: search on the lists of references and through contact with experts. Using these methods, two other studies were included.

### Study Characteristics

The included articles were composed by 187 participants and were classified as cross-sectional studies according to the JBI Manual. They were carried out in four countries, i.e., The Netherlands (Van Maarseveen et al., 2018), Portugal (Batista et al., 2019), United States (Brobst and Ward, 2002), and Germany (Brandes and Elvers, 2017; Low et al., 2021, 2022). Two studies included only female players (n = 17) (Brobst and Ward, 2002; Van Maarseveen et al., 2018), and five studies included only youth soccer players (Brandes and Elvers, 2017; Brobst and Ward, 2002; Low et al., 2021, 2022; Van Maarseveen et al., 2018). Only one study included adult players who competed at a semi-professional level (Batista et al., 2019).

The instructional constraints used in the considered studies were observed at three different moments: before (Batista et al., 2019; Brobst and Ward, 2002; Low et al., 2021, 2022), during (Brandes and Elvers, 2017) and after the practice or game (Brobst and Ward, 2002; Van Maarseveen et al., 2018). In studies in which the instructional constraint was performed before the game, the first considered three scenarios: without instruction, instruction about defense, and instruction about attack (Batista et al., 2019), the second used instruction related to defense strategy: high-press and deep defending (Low et al., 2021), and the last used instructions concerned the tactical formation applied by defending teams (Low et al., 2022). The study that used instructional constraints during the game applied two types of feedback: strongly pushed and unobstructive feedback (Brandes and Elvers, 2017), and in the study that used instructional constraints after the game, video-feedback was used (Van Maarseveen et al., 2018). One of the studies considered the feedback at two moments, with intervention consisting of the establishment of a goal that should be accomplished by the player (before), verbal feedback and the public posting of players’ performance (after) (Brobst and Ward, 2002).

Three studies assessed players’ tactical behavior using positional data (Batista et al., 2019; Low et al., 2021, 2022). The other three studies used notational analysis, one using a validated instrument (Team Sports Assessment Procedure, TSAP) (Brandes and Elvers, 2017) and the last two using subjective analysis, based on the knowledge of an experienced coach (Brobst and Ward, 2002; Van Maarseveen et al., 2018). Table 1 presents more details about the included studies.

**Table 1:** Characteristics of studies.

Authors (year)		Country		Sample		Context		Feedback type		Feedback moment		Game format		Measure of tactical behavior		Primary outcomes	
Brobst and Ward (2002)		United States		3 female players aged 15–17 years		High school soccer team; players had at least 5 years of experience in soccer		1) feedback on performance; 2) goal setting; 3) public posting of the player performance		Before and after		Friendly games		Notational analysis. Players’ behavior during friendly games was compared with “appropriated movements”		The intervention seemed to be effective in improving performance during friendly games.	
Brandes and Elvers (2017)		Germany		16 male players aged (mean) 17.2 years (SD = 0.7)		U-19 team playing the second division in Germany		Strongly pushed feedback vs. Unobtrusive feedback		During		SSG 4 vs. 4 + GK, 3 x 4 min with 2 min of passive rest		Notational analysis using the Team Sports Assessment Procedure (TSAP)		All game performance^1^ measures decreased under the strongly pushed condition.	
Van Maarseveen et al. (2018)		The Netherlands		14 female players with the mean age of 15.8 years (SD = 1.3)		Players from a national soccer talent team with 9.5 years (SD = 2.6) of experience in soccer		Video-feedback about the performance		After		SSG 3 vs. 3(2+GK). In each session, players participated in 12 attacking trials		Notational analysis using the performance score that was judged by the coach		No significant beneficial effect on the performance was observed, but it can be used to enhance the players’ understanding of correct tactical behaviors during training.	
Batista et al. (2019)		Portugal		16 male players with the mean age of 23.9 years (SD = 5.4)		Adult team playing at a semi-professional level		Without instruction vs. instruction about defense vs. instruction about offense		Before		SSG 7 vs. 7 + GK, 2 x 5min with 3 min of passive rest		Positional data analysis		The coaches’ instruction seemed to constrain players’ tactical behavior when compared with the without-instruction condition.	
Low et al. (2021)		Germany		69 male players with the mean age of 16.2 years (SD = 0.8)		Three U-17 teams from Germany. Players had 10.1 years (SD = 2.4) of experience in soccer		Instruction about which type of defense (high press vs. deep defending) should be used to recover the ball		Before		Trial using an official pitch, 10 vs. 10 + GK, following official soccer rules. Each trial ended when a goal was scored, the ball was conquered or the game was stopped		Positional data analysis + passing network analysis		Depending on the defensive instruction given by the coach, differences were observed in collective tactical behavior, showing better values to high press defending strategy.	
Low et al. (2022)		Germany		69 male players with the mean age of 16.2 years (SD=0.8)		Three U-17 teams from Germany. Players had 10 years (SD = 2) of experience in soccer		Instruction about which tactical formation (4-4-2 vs. 5-3-2) should be used to recover the ball		Before		Trial using an official pitch, 10 vs. 10 + GK, following official soccer rules. Each trial ended when a goal was scored, the ball was conquered or the game was stopped		Positional data analysis + passing network analysis		Depending on the team formation instruction given by the coach, differences were observed in collective tactical behavior, showing that defending in 5-3-2 led to reduced distances between players compared to 4-4-2.	
Low et al. (2021)		Germany		69 male players with the mean age of 16.2 years (SD = 0.8)		Three U-17 teams from Germany. Players had 10.1 years (SD = 2.4) of experience in soccer		Instruction about which type of defense (high press vs. deep defending) should be used to recover the ball		Before		Trial using an official pitch, 10 vs. 10 + GK, following official soccer rules. Each trial ended when a goal was scored, the ball was conquered or the game was stopped		Positional data analysis + passing network analysis		Depending on the defensive instruction given by the coach, differences were observed in collective tactical behavior, showing better values to high press defending strategy.	
Low et al. (2022)		Germany		69 male players with the mean age of 16.2 years (SD=0.8)		Three U-17 teams from Germany. Players had 10 years (SD = 2) of experience in soccer		Instruction about which tactical formation (4-4-2 vs. 5-3-2) should be used to recover the ball		Before		Trial using an official pitch, 10 vs. 10 + GK, following official soccer rules. Each trial ended when a goal was scored, the ball was conquered or the game was stopped		Positional data analysis + passing network analysis		Depending on the team formation instruction given by the coach, differences were observed in collective tactical behavior, showing that defending in 5-3-2 led to reduced distances between players compared to 4-4-2.	

^1^ Game performance measures: the number of conquered balls, the number of received balls, the number of lost balls, the number of neutral balls, the number of passes and the number of successful shots on the goal

### Risk of Bias and Confidence in Cumulative Evidence of Studies

Following the selected tool to evaluate the risk of bias (JBI checklist) of the included articles, three of them met 7 of the 8 items proposed by the instrument, one met 6 of the 8 items, one met 5 of the 8 items, and one met only 2 of the 8 items. Considering the first item, all articles were not clear in describing the inclusion criteria. Two studies did not use a valid and reliable way to measure the tactical behavior, considering the coach’s subjective analysis (Brobst and Ward, 2002; Van Maarseveen et al., 2018). In Table 2, an overview of the assessment of the risk of bias in individual studies can be seen.

**Table 2:** Assessment of the risk of bias in individual studies.

Authors	Q1	Q2	Q3	Q4	Q5	Q6	Q7	Q8	Quality assessment
Brobst and Ward (2002)	U	✓	--	✓	--	--	--	--	2
Brandes and Elvers (2017)	U	✓	✓	✓	✓	✓	✓	✓	7
Van Maarseveen et al. (2018)	U	✓	--	✓	✓	✓	✓	✓	6
Batista et al. (2019)	U	✓	✓	✓	--	--	✓	✓	5
Low et al. (2021)	U	✓	✓	✓	✓	✓	✓	✓	7
Low et al. (2022)	U	✓	✓	✓	✓	✓	✓	✓	7

Q1. Were the criteria for inclusion in the sample clearly defined? Q2. Were the study subjects and the setting described in detail? Q3. Was the exposure measured in a valid and reliable way? Q4. Were objective, standard criteria used for measurement of the condition? Q5. Were confounding factors identified? Q6. Were strategies to deal with confounding factors stated? Q7. Were the outcomes measured in a valid and reliable way? Q8. Was appropriate statistical analysis used? ✓: Yes; --: No; U: Unclear; N/A: Not/Applicable (Moola et al., 2020)

With the use of an adapted version of the quality index (Downs and Black, 1998), it was observed that out of the six studies included in the systematic review, four met 10 of the 14 items used (71.42%) (Brandes and Elvers, 2017; Low et al., 2021, 2022; Van Maarseveen et al., 2018), while Brobst and Ward (2002) met 7 items (50%), and Batista et al. (2019) met 9 items (64.28%). Table 3 presents more information about the assessment.

**Table 3:** Critical appraisal of studies included in the systematic review

Authors (year)		Criteria																
		1^st^	2^nd^	3^rd^	6^th^	7^th^	10^th^	11^th^	12^th^	15^th^	16^th^	18^th^	20^th^	22^nd^	23^rd^	n.	%
Brobst and Ward (2002)		1	1	1	1	0	0	0	0	0	1	1	0	1	0	7	50.00
Brandes and Elvers (2017)		1	1	1	1	1	1	0	0	0	1	1	1	1	0	10	71.42
Van Maarseveen et al. (2018)		1	1	1	1	1	1	0	0	0	1	1	0	1	1	10	71.42
Batista et al. (2019)		1	1	1	1	1	0	0	0	0	1	1	1	1	0	9	64.28
Low et al. (2021)		1	1	1	1	1	1	0	0	0	1	1	1	1	0	10	71.42
Low et al. (2022)		1	1	1	1	1	1	0	0	0	1	1	1	1	0	10	71.42
Total	n.	6	6	6	6	5	4	0	0	0	6	6	4	6	1		
	%	100.00	100.00	100.00	100.00	83.33	66.66	00.00	00.00	00.00	100.00	100.00	66.66	100.00	16.66		

1^st^: Is the hypothesis/aim/objective of the study clearly described? 2^nd^: Are the main outcomes to be measured clearly described in the Introduction or Methods section? 3^rd^: Are the characteristics of the participants included in the study clearly described? 6^th^: Are the main findings of the study clearly described? 7^th^: Does the study provide estimates of the random variability in the data for the main outcomes? 10^th^: Have current probability values have been reported (e.g., 0.035 rather than <0.05) for the main outcomes except where the probability value was less than 0.001? 11^th^: Were the subjects asked to participate in the study representative of the entire population from which they were recruited? 12^th^: Were those subjects who were prepared to participate representative of the entire population from which were they recruited? 15^th^: Was an attempt made to blind those measuring the main outcomes of the intervention? 16^th^: If any of the results of the study were based on “data dredging”, was this made clear? 18^th^: Were the statistical tests used to assess the main appropriate outcomes? 20^th^: Were the main outcome measures used accurate (valid and reliable)? 22^nd^: Were study subjects in different intervention groups (trials and cohort studies) or were the cases and controls (case-control studies) recruited over the same period? 23^rd^: Were study subjects randomized to intervention groups? 0: No/Unable to determine; 1: Yes (Downs and Black, 1998)

### Results of Individual Studies

In this section, we will focus on the tactical behavior and its interaction with any type of instructional constraint. It should be noted that some of the investigated studies applied protocols that assessed different performance variables.

Brobst and Ward (2002) investigated the effects of public posting, goal setting, and oral feedback intervention during a season on the ball-handling skills with three female players from a high school soccer team. They also sought to assess how to generalize these effects to the game, and to measure the acceptability of the intervention by coaches and players. The study was conducted during the season which consisted in 27 training sessions and 10 games, and was divided in three moments: baseline, intervention, and maintenance. The players’ tactical performance was evaluated using three variables: movements with the ball, movements during restarts, and movements after the player passed the ball. These variables were coded as appropriate when correctly executed, and inappropriate otherwise, by two soccer players from the adult league, and by the first author. As feedback intervention, three strategies where used: public posting, goal setting, and verbal feedback. The first one was a chart displayed to the whole team with the percentage of adequate performance, the second was the setting of the goal that needed to be accomplished by players, and the last one was verbal feedback given by the coach about the players’ goals and performance. In the maintenance phase, it was observed that players continued to perform the movement with the ball at a good level, while the other two behaviors decreased in quality. In general, the result showed improvement in the percentage of adequate behaviors during friendly games, which cannot, however, be generalized to official games.

The purpose of Brandes and Elvers (2017) was to determine the impact of mildly vs. strongly pushed coach feedback on the physiological response, the ratio of perceived exertion, time-motion characteristics, and game performance in soccer training with small-sided games (SSGs), of sixteen youth players belonging to an U-19 team who played in the German second division. In that study, feedback within 4 vs. 4 SSGs was used, modulating the way and frequency that the intervention was carried out with each of the groups. Under the strongly pushed feedback condition, the strategy was to speak loudly and constantly, encouraging the team to put pressure on the opponent to score a goal or to return to defense to recover the ball, evaluating each player’s action. Under the unobtrusive feedback condition, the coach only performed motivational interventions through encouragement, without directly defining what actions players should perform. They played two 4 vs. 4 SSGs each, and during the game, the coach provided mild, unobstructive or strongly pushed feedback. Regarding the tactical characteristics observed in the study, the Team Sports Assessment Procedure (TSAP) (Gréhaigne et al., 2012) was used to evaluate game performance, which consisted of six tactical actions: the number of conquered balls, the number of received balls, the number of lost balls, the number of neutral balls, the number of passes, and the number of successful shots on the goal. It was found that in SSGs with strongly pushed feedback, all game performance measures were possibly or likely to decrease, but did not reach the statistical significance level.

Van Maarseveen et al. (2018) evaluated the effects of self-controlled feedback on tactical skills in small-sided soccer games of fourteen youth female players from the Netherlands, in which the experiment consisted in a pretest, a training period and a post-test. The study subjects played 3 vs. 2. SSGs (3 attackers versus 2 defenders and a goalkeeper). In total, seven training sessions were performed, and in each training session, players participated in 12 attacking trials. In the pretest and post-test, players participated in 15 attacking trials, and their tactical performance was recorded and then evaluated by an experienced coach. Regarding the video-feedback used in the study, every two attempts, players received feedback. Two groups were defined: a self-control group could decide from which trial the video-feedback was provided, and the other received video-feedback on the same schedule of the self-control group. The three attackers and the coach watched the video-feedback together, and the conversations around the feedback session were recorded to evaluate the frequency of conversations about negative, neutral, and positive aspects of the performance or improvements. As the main results, no significant differences in performance between pre- and post-test in any of the groups were observed, and the feedback request was more frequent after a good trial. Regarding conversations about the video-feedback, it was observed that the coach played a major role in these moments, but players of the self-control group showed more initiative than the control group. Despite these findings, no effects on tactical behavior were observed.

Batista et al. (2019) evaluated the effects of previous instruction on technical, tactical and external workload performances in small-sided soccer games of sixteen adult semi-professional male players. Two teams composed of seven players each, played 7 vs. 7 games, twice per day on two different days, in a randomized sequence regarding the instruction given to athletes: instruction about defense (DS), instruction about offense (OS), and without instruction (WSI). Using positional data, the study assessed three tactical behavior variables: team length, team width, and effective playing space. Analyzing these conditions, lower effective playing space for DS in comparison with WSI was observed, as well as higher team length and higher effective playing space for OS in comparison with WSI. Thus, it was observed that the coaches’ instruction constrained players’ tactical behavior when compared to the condition without instruction.

In the study of Low et al. (2021), the aim was to analyze players’ tactical behaviors from their positional data, as an effect of two contrasting pressing strategies, high-press defending and deep defending. Sixty-nine soccer players from three different under-17 clubs of Germany participated in the study. The trial-based experimental approach consisted of 11 vs. 11 games, played on an official-sized pitch, in which each team performed six trials of repeated measures for each condition, resulting in 72 trials (36 trials per condition). The attacking team received the following instruction: “score a goal”, and the defending team was instructed to “win the ball using a high-press defending strategy” or “win the ball using a deep-defending strategy”. To assess behaviors during trials, measures in different scales were used: inter-team distance, and trial length (s) at the game level, distance to the nearest opponent, dyadic distance, team length, team width, and length per width at the team level, defense-midfield distance, midfield-forward distance, and defense-forward distance at the group level, the center midfield left area, the center midfield right area, the attacking midfielder area, and the forward area at the individual level. At the team level, the space control gain for the attacking team was also measured. In addition, they performed a passing network analysis. Depending on the instruction, the players’ behavior changed, and it was observed that when defending using the high-press strategy, the distances between teams were closer, and players’ dispersion was larger due to the longer team length. For the attacking team, the ball possession time was reduced, the area occupied by midfielders and forwards was larger, and more penetrative passes were performed. In conclusion, players seemed to follow the instruction given by the coach, modifying their behaviors according to the strategy adopted.

Low et al. (2022) aimed to examine the tactical behaviors of 69 players, modulating through the coach instruction the defending formations (4-4-2 and 5-3-2), in 11 vs. 11 games. The attacking team played in the 4-3-2-1 formation, and had as a task to score a goal, while the defending team had to win the ball using a 4-4-2 or a 5-3-2 formation, depending on the coach instruction. To assess the tactical behaviors, positional data were used, gathered using the GPS, and then chosen metrics were used to evaluate these behaviors: inter-team distance and trial duration (game level), distance to the nearest opponent, dyadic distance, team length, team width, length per width ratio, space control gain (team level), inter-line distance (group level), distance to each opponent (dyadic level), and individual area (individual level). To complement the analysis, passing networks were also assessed through notational analysis. From the 72 trials registered in the study, small differences between the two conditions were observed. When compared to defending in the 4-4-2 formation, defending in the 5-3-2 formation decreased the team dispersion, distances between midfielders and forwards and between the defender and forwards. The team width was reduced to the attacking team, and considering the passing network analysis, minor need to make passes to the goalkeeper was observed. Manipulating the defensive strategy seemed to constrain the behaviors of the opposing team.

## Discussion

This review aimed to investigate the role of instructional constraints performed by coaches on players’ tactical behavior. To do so, the emphasis was on the deliberate practice environments, with soccer players at any performance level. It was found that instructional constraints, given as instruction (before the game or training sessions) had greater effect on players’ tactical behavior when compared with feedback (during or after).

Regarding the protocols of studies, some methodological differences were observed in terms of coaching strategies, and the measures used to assess tactical behaviors. These reasons did not allow us carrying out a meta-analysis, thus pointing out the need for more research on this topic, and demonstrated the importance of considering valid and reliable instruments to evaluate tactical behavior. The results showed that players’ behavior was altered by the instructional constraints depending on the type and the moment of these interventions. When the intervention was performed before training or game, as an instruction, players seemed to have more clarity about how to coordinate their collective actions, while the individual feedback did not bring clear benefits to these collective behaviors. Nonetheless, readers need to be cautious when interpreting the findings of studies included in this review, considering the flaws and risk of bias observed, which can lead to misinterpretation of interventions and results. Regarding the secondary goal of this review evaluating the theoretical approach of studies, only few provided clues on the topic and, to avoid a subjective analysis, such assessment was not conducted.

### Instructional Constraints

In the study by Brobst and Ward (2002), three different instructional constraints were used: verbal intervention on performance (given after training), public posting, which consisted of presenting the development of players’ performance in the form of graphics (given after training), and through the stipulation of objectives that should be achieved by players throughout the season (given before training). The study showed improvement in the young women’s performance. However, we were unable to establish the effects of each of them or judge them as effective, since they were applied together, in addition to the fact that the improvement in performance may have been the result of training, but due to the absence of a control group for comparison purposes, the results became biased.

Brandes and Elvers (2017) compared two different types of feedback strategies. The strongly pushed feedback strategy showed a decrease in all game performance measures, which corroborated studies that indicated that the excessive and continuous use of instructional constraints of the instructive/direct type could delay the processes of internalizing information, in addition to generating an environment of pressure that could be harmful to less experienced players, limiting their performance (Otte et al., 2020). Van Maarseveen et al. (2018), in small-sided 3 vs. 3 games, used video feedback to provide information about players’ performance. During the moments that videos were presented, the coach and players discussed the observed situations; however, the content of these interactions was not presented in the study. The study carried out a word count to verify the frequency of participation of the coach and players during conversations, in which predominance of coach’s speeches was observed, and in the self-controlled group, this predominance decreased. Furthermore, it was verified that the self-controlled group preferred asking for feedback after good executions, as a way of confirming the success of that attempt.

The other three studies included in the review (Batista et al., 2019; Low et al., 2021, 2022) bring different aspects related to instructional constraints before the games. Batista et al. (2019), in small-sided 7 vs. 7 games, used three conditions: without an instruction, with a defensive instruction, and with an offensive instruction, while Low et al. (2021) used instructions regarding the defensive strategy that the team should use to recover ball possession, i.e., high pressing or lower defense; and Low et al. (2022) modulated the type of the tactical formation that the defending team should use to recover ball possession: 4-4-2 vs. 5-3-2, in both studies, games were played on an official soccer field and had an 11 vs. 11 format. In those studies, the instruction given before the games constrained players’ behaviors, because players tended to execute actions according to the coach’s strategy. Since soccer is a collective game, it is reasonable to state that individual actions reflect on group behaviors. In this way, by creating behavioral patterns for players, giving instructions on how to defend, attack or position themselves into the game pitch, players might have better ideas about how to play in the game. Otherwise, when feedback is directed at an individual scale, this coordination of actions became more difficult. Furthermore, indirect influence of the coach’ instructions on the opposite team’s tactical behavior is observed, which can emerge from the continuous exchange of information among both teams. These interactions appear by observing how the opposing team occupies space and carries out their passing dynamics.

### Measures of Tactical Behavior

Before discussing the types of measures adopted to evaluate tactical behavior, it is important to highlight in what environments these behaviors were carried out, since variations in game formats and conditions can induce different tactical, technical, physical and physiological responses (Praça et al., 2022), and the manipulation of functional and structural constraints of the task leads to the emergence of new tactical behavior patterns, as well as interactions among players (Ometto et al., 2018; Ueda et al., 2023). Among studies included in this review, three of them used small-sided games to carry out interventions, namely a) 4 vs. 4 with GK in games lasting 3 x 4 min, with a field size of 40 x 40 m (Brandes and Elvers, 2017), b) 3 vs. 2 + GK in games with duration determined by the task rule (when the ball went out of lines or a goal was scored, the attempt was over), with a field size of 40 x 25 m (Van Maarseveen et al., 2018), and c) 7 vs. 7 with GK in games lasting 2 x 5 min, with a field size of 62 x 50 m (Batista et al., 2019). One of the articles did not define the size of games or the rules applied, classifying the intervention as practice scrimmages (Brobst and Ward, 2002). The last two studies had the same type of intervention, considering 11 vs. 11, played on an official-sized field, which ended when a goal was scored, the ball was recovered by the defense or the game stopped for some other reason (Low et al., 2021, 2022). This description is important, as the instructional constraint used must consider not only the characteristics and performances of players but is also related to the environment and the task in which the game is carried out.

Regarding tactical behavior, it is a fact that the coach’s knowledge is essential for the best provision of information to players in order to help them in the search for the best answers to the presented problems (Otte et al., 2020; Sigrist et al., 2013), but when considering the evaluation or measurement of a construct, it is important to use validated and reliable instruments. Therefore, when measuring the players’ tactical behavior based on the coach’s observation and subjective assessment, the risk of bias increases (Aromataris and Munn, 2020). The methods used in the studies included in this review can be divided into two categories: notational analysis and positional data analysis. Studies that used notational analysis were divided into analyzing through a validated instrument (Brandes and Elvers, 2017), and through subjective evaluation, considering the coach’s knowledge to classify players’ tactical behaviors (Brobst and Ward, 2002; Van Maarseveen et al., 2018), and in all these scenarios, observation was carried out at an individual level. The other three studies used data extracted from the GPS (global position tracking systems), using a series of metrics calculated from the variation in the player’s movements at different organizational scales; two of them carried out a complementary analysis using passing networks (Low et al., 2021, 2022).

Regarding studies that used notational analysis, the study by Brobst and Ward (2002) showed movements in players with the ball, movements during game restarts and movements after passing the ball, and compared these movements with expected patterns, previously defined by the coach. In the study by Van Maarseveen et al. (2018), an experienced coach evaluated each attacker’s tactical behavior, considering actions with the ball and positioning on the field, giving a score from 1 to 10 in each attempt made by players; however, the criteria used were not informed. Finally, Brandes and Elvers (2017) used a validated instrument named Team Sports Assessment Procedure (TSAP) proposed by Gréhaigne et al. (2012), in which the number of balls recovered, the number of balls received, the number of balls lost, the number of neutral balls, the number of passes and the number of correct shots at the goal were individually observed, being an analysis with tactical and technical characteristics. Regarding the collective analysis of the tactical behavior of soccer players, the use of GPS positional data is a good alternative, as it allows assessing movement variations over time. Using this instrument, Batista et al. (2019) evaluated three metrics related to tactical behavior at a collective level: width, length and effective playing space. Low et al. (2021, 2022) evaluated the same construct considering the individual, dyadic, group, collective and game scales. At an individual level, the area occupied by the player was observed. At a dyadic level, the distance between these two elements was observed. At a group level, the distance between groups of players was observed, and at a team level, the occupation of the game space regarding width, depth and distances to opponents was analyzed. Finally, at a match level, the average distance between the two teams was considered.

In summary, considering the types of instructional constraints presented by studies included in this review, those given before training have greater effect on players' tactical behavior (Batista et al., 2019; Low et al., 2021, 2022), when compared to interventions during or after training. A possible explanation for this result is that players tend to follow, at least partially, the strategies proposed by the coach, which affects the way they position themselves and move on the field; in other words, players have more clarity on how to coordinate their actions (how to defend, how to attack, where to be in the field, etc.) to achieve the proposed goals. Furthermore, the findings shed light on the common practice of offering information throughout games, which according to results, may not be the best strategy adopted by coaches to help players coordinate their actions. Finally, regarding feedback related to tactics, even if it is directed at a single individual, it is important to understand that its effect tends to spread throughout the system; therefore, when evaluating these effects, it is necessary to consider a larger scale for observing the construct.

### Practical Implications and Study Limitations

This systematic review provides evidence that allows a better understanding of the observed phenomena and brings to readers an overview of studies on the topic of instructional constrains and tactics. In general, coaches and other individuals interested in soccer and other sports can rely on organized information to improve their professional practice. Understanding the types of interventions that influence players’ behaviors can be valuable to both coaches and methodological coordinators, in the search for better structuring of the environment and training practices.

This study has two main limitations. The first is related to the heterogeneity of studies, regarding the type of interventions and the way of evaluating tactical behavior, not allowing a direct comparison between them. The second limitation is related to the low number of studies, not allowing the generalization of observed results and preventing the evidence from being synthesized to answer the question that guided the review.

Future studies should make use of validated instruments to evaluate tactical behavior, objectively present the type of perspective used in the study and more clearly describe the intervention carried out by coaches, bringing the content present in these interventions. Since the included studies applied their interventions only during training sessions, future studies may consider investigating coaches’ instructional constraints during actual matches. Furthermore, greater accuracy when reporting each stage of studies should be achieved, so that the methods used are more clearly translated for the reader.

## Conclusions

First, it is possible to observe that the instructional constraints used by coaches before training play the role of providing better insights for players on how to coordinate their actions, which has direct influence on their tactical behaviors. Considering one of the most important characteristics of soccer, the fact that it is a collective game, it is crucial to have in mind that collective organization is important to coordinate the players’ action in the game. Coaches play the role of guiding players to that level of organization, and to do so, they can manipulate some aspects of the training environment. The manipulation of the proposed tasks could present problems to be solved by players, and this search to solve problems can be facilitated with high-quality information provided by an external agent. However, this information should help, not limit, the player’s search. In this sense, players need to have the freedom to explore the relationships created within the game space, since interventions where coaches speak loudly and constantly showed negative effects on the players’ performance. Finally, in a system in which all elements interact and influence each other, it is important to evaluate the construct with instruments that demonstrate this interaction, observing the phenomena in a higher scale.
